# Bilateral Idiopathic Pyoderma Gangrenosum: A Case Report of an Atypical Presentation

**DOI:** 10.1002/ccr3.71964

**Published:** 2026-01-28

**Authors:** Sanjog Thapa Magar, Deekshanta Sitaula, Subi Rijal, Vikash Paudel, Bhaskar M. M. Kayastha

**Affiliations:** ^1^ Department of Dermatology and Venereology Patan Academy of Health Sciences Lalitpur Nepal

**Keywords:** autoimmune diseases, neutrophil disorders, pyoderma gangrenosum, skin ulcer, wound healing

## Abstract

Pyoderma gangrenosum (PG) is a rare non‐infectious neutrophilic dermatosis which is characterized by a rapidly progressive, painful ulcer. Bilateral manifestation of PG is exceptionally rare and can easily be misdiagnosed as infection or vascular ulceration, delaying proper treatment. A 76‐year‐old woman presented with painful, crusted ulcers on her bilateral lower legs that developed over 2 weeks. Initially, the lesions erupted as erythematous papules that enlarged rapidly and ulcerated. There was no history of trauma, systemic illness, or prior ulceration. Laboratory tests revealed mild anemia and an elevated C‐reactive protein level. Swab culture from the wound site showed growth of *Pseudomonas aeruginosa* . There was no response to antibiotics, suggesting a non‐infective etiology. An incisional skin biopsy revealed dense neutrophilic infiltrate with dermal necrosis in the absence of vasculitis or infection, findings characteristic of neutrophilic dermatosis and consistent with PG. The patient was treated with oral prednisolone and local saline dressings, resulting in significant improvement within 2 weeks and complete healing without recurrence. PG can occur even in the absence of systemic diseases and may mimic infectious or vascular ulcers. Awareness about atypical presentations, early biopsy and timely initiation of corticosteroid therapy is essential to avoid misdiagnosis and achieve favorable outcomes.

## Key Clinical Message

Bilateral involvement of pyoderma gangrenosum is extremely rare and can easily be misdiagnosed as infection or vascular ulcers. Early biopsy, exclusion of other similar conditions, and rapid initiation of corticosteroid therapy are essential to prevent unnecessary interventions and achieve favorable outcomes.

## 1 Introduction

Pyoderma gangrenosum (PG) is a rare, autoinflammatory neutrophilic dermatosis first described by Brunsting et al. in 1930 [1]. Its estimated annual incidence is very low, ranging between 3 and 10 cases per million population [2]. It usually appears as a rapidly enlarging, very painful ulcer with violaceous, undermined edges, most often affecting the lower legs. PG is associated with systemic conditions in up to 75% of cases, most often inflammatory bowel disease, inflammatory arthritis, or hematologic malignancy [3]. As there is no single diagnostic test, establishing the diagnosis is largely a matter of clinical judgment and exclusion of other causes such as infection, vasculitis, neoplasia, or vascular ulcers [3, 4].

Among the recognized clinical variants, the ulcerative type is by far the most frequent, although pustular, bullous, and vegetative forms have been described [3, 5]. Lesions are typically unilateral, most commonly localized to a single pretibial area [6]. Bilateral involvement is exceedingly uncommon and can easily mislead clinicians toward more common diagnoses like cellulitis, necrotizing infections, or vascular disorders [5]. Only a small number of bilateral PG cases have been published, and these often present significant diagnostic challenges [7, 8].

We present here the case of an elderly woman who developed bilateral ulcerative PG without any associated comorbid conditions. She responded well to systemic corticosteroid therapy combined with supportive wound care. In the discussion, we compare this case with recently published reports in order to identify shared features, important differences, and practical lessons for clinicians.

## 2 Case Report

### 2.1 Case Presentation

**FIGURE 1:**
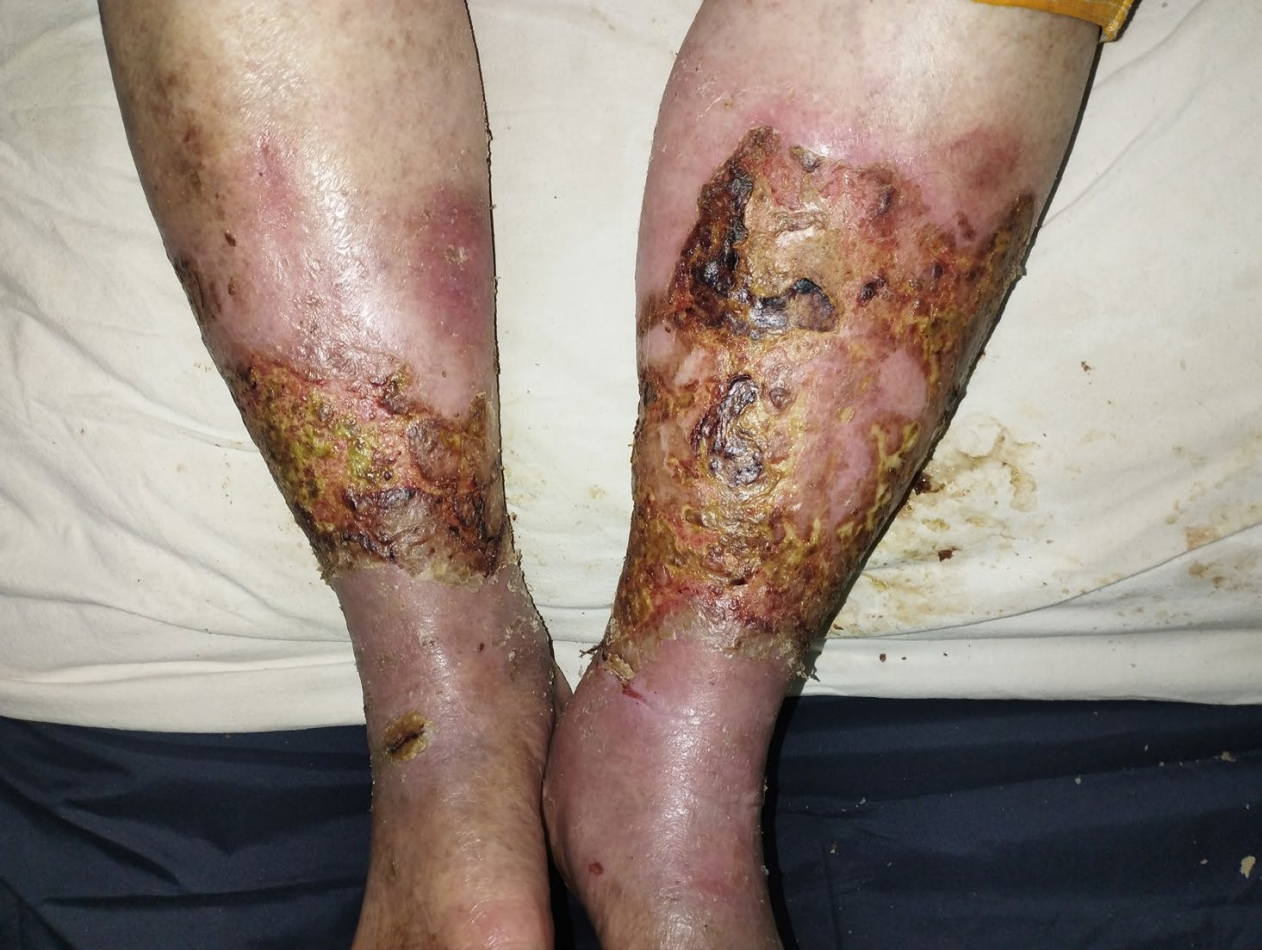
Extensive ulcerative, necrotic, and crusted lesions on both lower legs consistent with active pyoderma gangrenosum.

A 76‐year‐old female presented with painful crusted lesions on both lower legs of 2 weeks' duration. Initially, the lesions appeared as erythematous papules that rapidly enlarged, subsequently oozed, and crusted (Figure 1). She denied any preceding trauma, systemic symptoms (fever, weight loss), or past history of ulcerations. She had no comorbidities such as diabetes, hypertension, autoimmune disease, inflammatory bowel disease, or malignancy and was not on any regular medications. On physical examination, multiple tender, ill‐defined, yellowish, and hemorrhagic crusted plaques with oozing and ulceration were seen on the bilateral shins and ankles, with surrounding erythema and edema. Peripheral pulses were intact; no regional lymphadenopathy was noted.

### 2.2 Investigation

**FIGURE 2:**
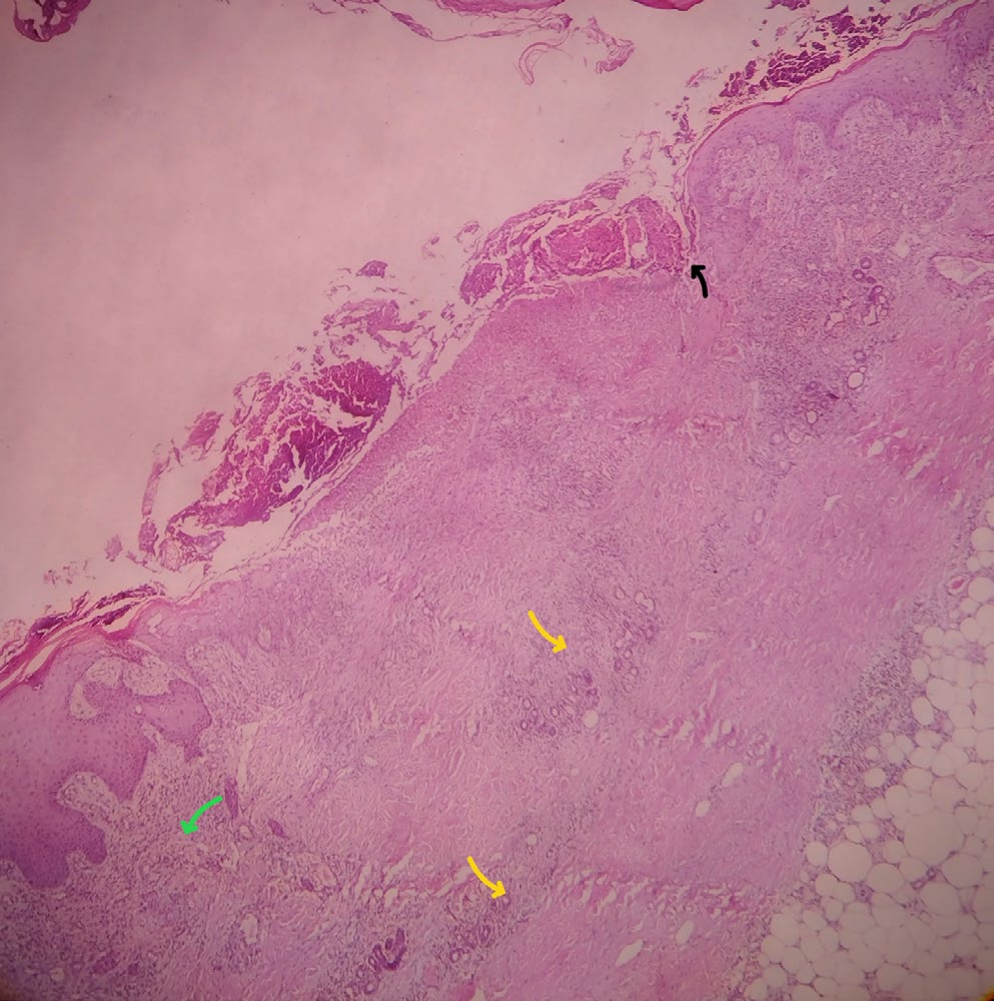
Superficial (green arrow) and deep (yellow arrow) dermal inflammation, ulceration (black arrow), and mixed inflammatory cell infiltrate, including neutrophils (H&E stain, low power magnification).

**FIGURE 3:**
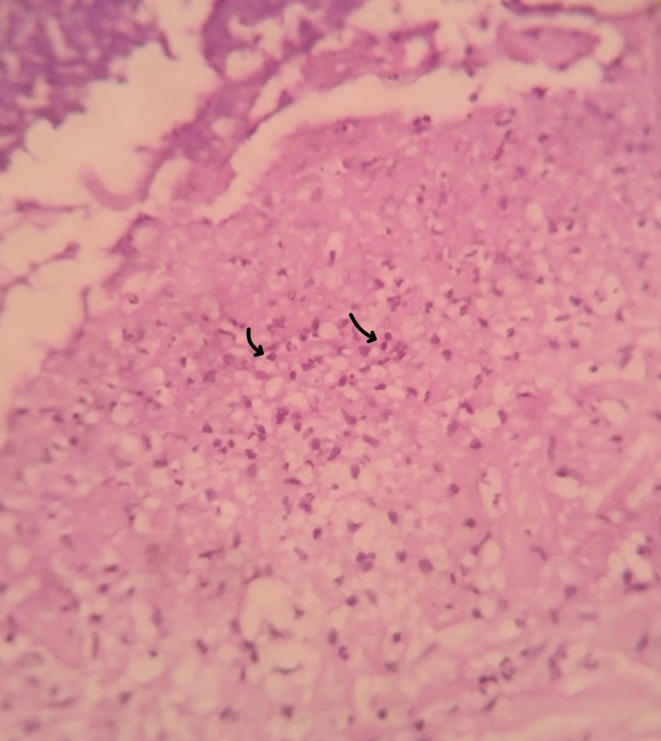
Dense neutrophilic infiltrate in the dermis (black arrows) with focal leukocytoclasia characteristic of pyoderma gangrenosum (H&E stain, high power magnification).

Laboratory work showed mild anemia and an elevated C‐reactive protein of 64.5 mg/L. A wound swab cultured *Pseudomonas aeruginosa* . Given the positive culture, infection was initially considered, and the patient was started on levofloxacin 500 mg orally once daily for 7 days but there was no clinical improvement despite appropriate antibiotic therapy. Although the wound culture grew *P. aeruginosa* , colonization is common in PG. In view of the absence of clinical response to adequate antibiotic therapy, the finding was considered colonization rather than the primary cause of the lesion and histopathological evaluation was sought. An incisional biopsy from the lesion edge revealed a dense neutrophilic infiltrate with dermal necrosis, without features of vasculitis, consistent with PG (Figures 2 and 3).

### 2.3 Diagnosis, Treatment and Outcome

**FIGURE 4:**
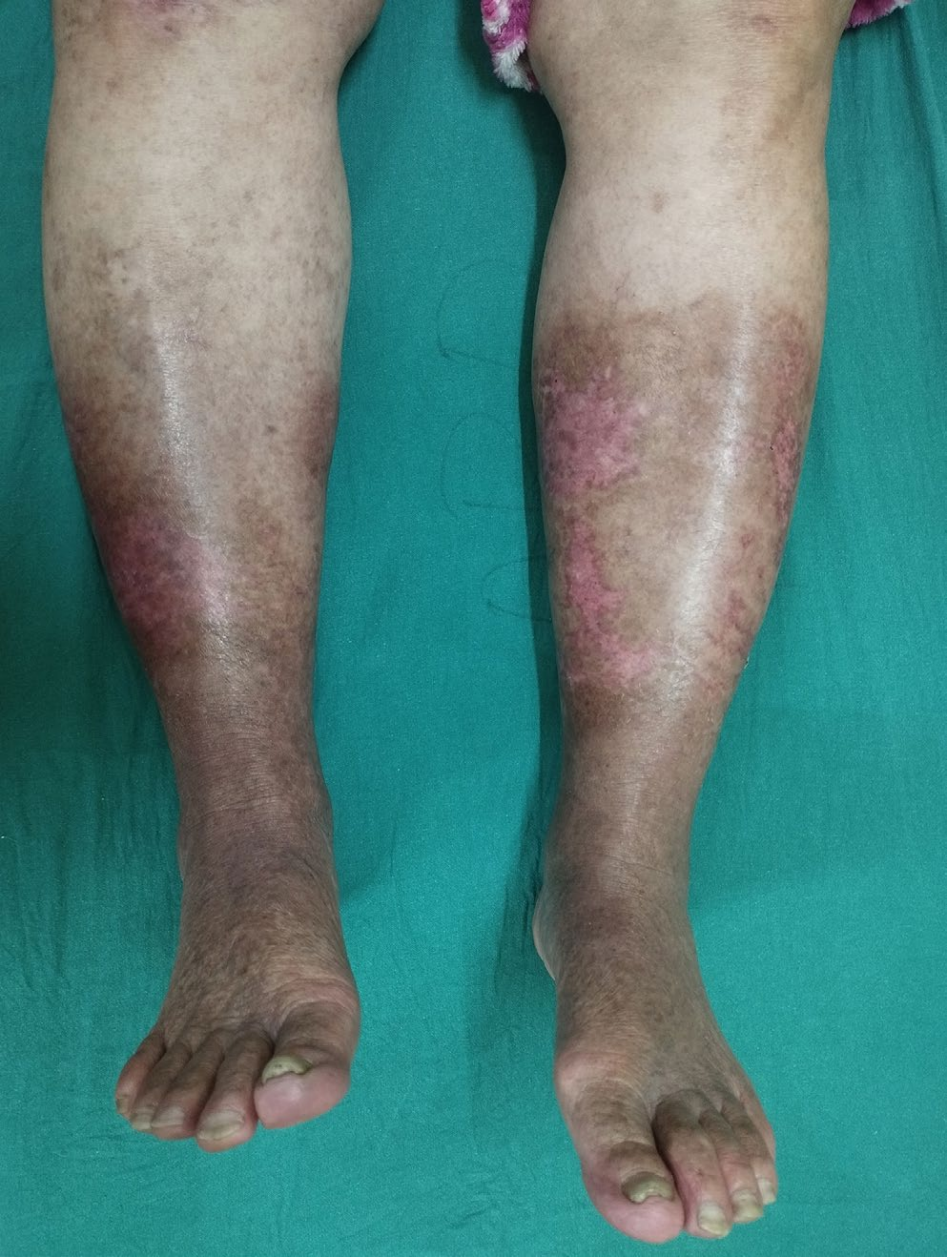
Healed skin with residual hyperpigmentation and scarring, showing resolution of pyoderma gangrenosum.

**FIGURE 5:**
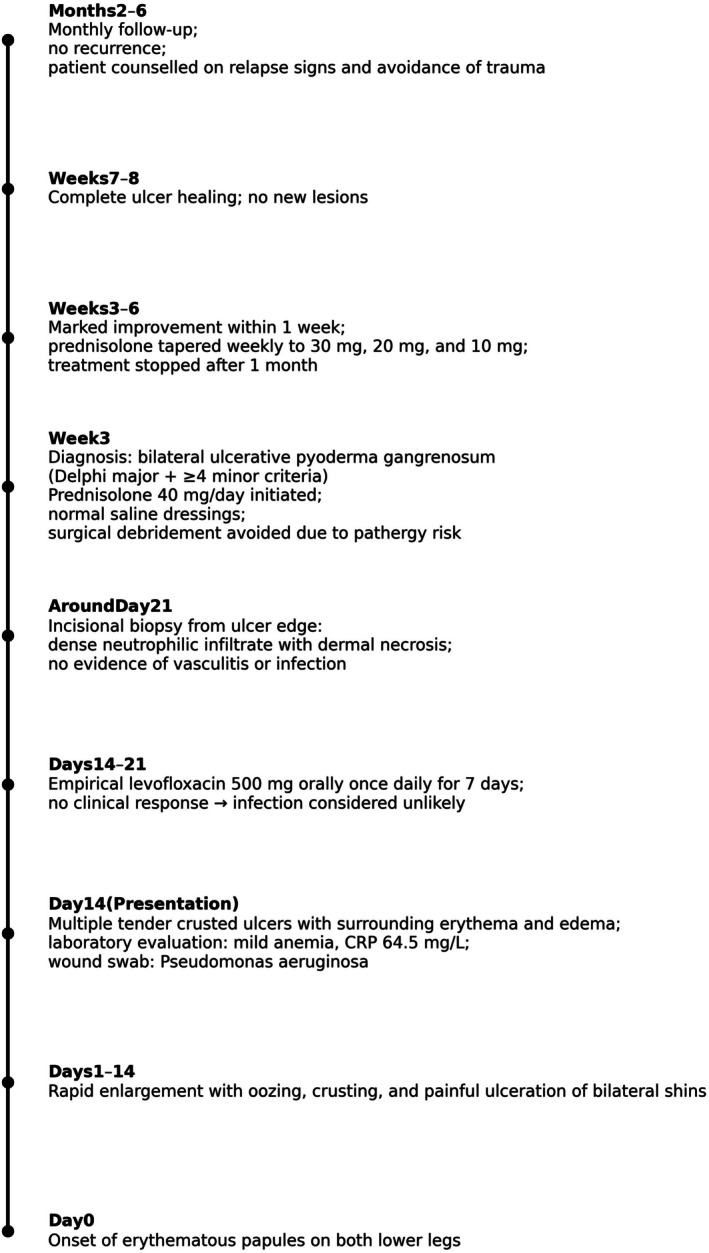
Timeline of clinical course, diagnosis, and treatment of bilateral ulcerative pyoderma gangrenosum.

This case fulfilled the Delphi consensus major criterion (neutrophilic infiltrate on biopsy) and multiple minor criteria for ulcerative PG. In particular, infection was reasonably excluded, the lesions began as rapidly ulcerating papules, there were ulcers over bilateral lower legs, the ulcers were painful with surrounding erythema and edema, and they showed clear improvement within 1 month of starting systemic corticosteroids. Together, these features satisfied the major criterion plus at least four minor criteria, supporting the diagnosis of ulcerative PG in this patient. Because of the risk of pathergy in PG, surgical debridement was deliberately avoided and the ulcers were managed conservatively with normal saline dressings alone. Within 2 weeks, there was significant ulcer healing (Figure 4). She was advised of monthly follow up for the next 6 months. During the follow‐up after a month, no recurrence was noted. The patient was counseled about the risk of PG relapse, instructed to seek prompt review for any new painful papules or ulcers, and advised on gentle skin care and avoidance of trauma to the lower limbs (Figure 5).

## 3 Discussions

PG is thought to result from dysregulated innate immunity, characterized by neutrophil overactivation, abnormal chemotaxis, and heightened proinflammatory signaling [3]. The diagnosis of PG is clinico‐pathologic and largely one of exclusion, with consensus guidelines such as the Delphi criteria providing structure [4]. As PG lesions are prone to secondary colonization, positive cultures often reflect colonization, not primary infectious etiology [9].

In this case, the lesions developed quickly over the course of 2 weeks, beginning as small erythematous papules that enlarged and formed painful, crusted ulcers on both shins. This rapid progression fits well with the acute neutrophilic activation described in the pathogenesis of PG [3]. Although *P. aeruginosa* was cultured from the wound, the absence of improvement with antibiotics and the biopsy findings of dense neutrophilic infiltration with dermal necrosis indicated an inflammatory rather than infectious cause. As no underlying systemic illness was identified, this case falls into the idiopathic group of PG, which represents about one‐third of all cases [3]. The bilateral distribution of the ulcers, however, is unusual and highlights how PG can deviate from its typical unilateral presentation, reminding clinicians to maintain a high level of suspicion when evaluating rapidly progressive leg ulcers.

### 3.1 Drug‐Induced/Biologic‐Related and Refractory PG

In our case, the 76‐year‐old woman in our case developed bilateral, rapidly progressive ulcers on the lower legs without any precipitating trauma or systemic illness, and her lesions healed quickly with oral prednisolone (40 mg/day) and saline dressings. In contrast, a 70‐year‐old woman developed PG while on anti‐IL‐17 therapy (Ixekizumab) and required IL‐36 receptor blockade (spesolimab) for remission in a previously published case report—highlighting how biologic therapy can trigger or unmask PG and how some cases demand cytokine‐targeted agents rather than standard corticosteroids [10]. Other reports have highlighted refractory PG managed with newer agents such as apremilast or spesolimab, illustrating how biologic and small‐molecule therapies are expanding the treatment options for difficult cases [10, 11]. Another case report also described PG developing in a patient receiving natalizumab, underscoring that immune‐modifying drugs themselves may precipitate PG—again differing from our patient, whose disease was idiopathic and responded well to first‐line therapy [12].

### 3.2 Post‐Operative and Malignancy‐Associated PG

In a previously reported postoperative case, abdominal‐wall PG developed following hysterectomy, bilateral salpingectomy, and omentectomy for stage IVB high‐grade serous carcinoma of the endometrium [13]. In patients with malignancies, PG may arise as a paraneoplastic or postoperative complication, driven by immune imbalance and tumor‐related cytokine activity that amplifies neutrophilic inflammation [14, 15].

### 3.3 Other Locations of PG and Misdiagnoses

Another case published in the *Journal of Inflammation Research* (2024) involved a 79‐year‐old man with facial PG that had initially been misdiagnosed as an infection; his ulcers resolved with steroids and cyclosporine. Like our patient, the lesion was painful and rapidly progressive, but the facial location and prolonged diagnostic delay set it apart [16]. A recently published case series of nine patients further supports the tendency for PG to be misidentified as infection or cellulitis, particularly when lesions occur on the lower limbs. Most of those patients were treated with systemic corticosteroids and wound care, and some required negative‐pressure wound therapy (NPWT) [17]. Our case paralleled this group in terms of lower‐limb site and initial diagnostic uncertainty, though the ulcers in our patient healed within 2 weeks and without NPWT, suggesting an unusually brisk response.

### 3.4 Overall Comparison

Overall, the recent literature shows that although PG typically presents with painful ulcers and a neutrophil‐rich inflammatory pattern, the underlying causes and treatment responses differ greatly from case to case. Our patient's presentation was unusual because the ulcers were bilateral, developed without any associated systemic disease, and responded rapidly to corticosteroid therapy. This contrasts with many published reports describing more complicated or treatment‐resistant disease. The comparison highlights that even with its classic features, PG can present in unexpected ways, and clinicians must stay alert to such variations. Timely biopsy, careful exclusion of infectious or vasculitic mimics, and early initiation of immunosuppressive therapy remain key to achieving good outcomes.

## 4 Conclusion

This case highlights an unusual bilateral presentation of PG in an elderly woman without any systemic disease, which responded rapidly to corticosteroid therapy. PG can occur even in the absence of systemic diseases and may mimic infectious or vascular ulcers. Recognizing such atypical presentations, performing an early biopsy, and initiating prompt immunosuppressive therapy are essential for achieving favorable outcomes and preventing unnecessary interventions.

## Author Contributions

**Sanjog Thapa Magar:** conceptualization, data curation, formal analysis, investigation, writing – original draft. **Deekshanta Sitaula:** conceptualization, data curation, formal analysis, investigation, writing – original draft, writing – review and editing. **Subi Rijal:** data curation, writing – original draft, writing – review and editing. **Vikash Paudel:** writing – original draft, writing – review and editing. **Bhaskar M. M. Kayastha:** writing – original draft, writing – review and editing.

## Funding

The authors have nothing to report.

## Consent

Written informed consent was obtained from the patient for publication of this case report.

## Conflicts of Interest

The authors declare no conflicts of interest.

## Data Availability

The authors have nothing to report.

## References

[1] L. M. Newell and F. D. Malkinson , ““Pyoderma (Ecthyma) Gangrenosum” by Brunsting, Goeckerman and O'leary, October 1930. Commentary: Pyoderma Gangrenosum,” Archives of Dermatology 118, no. 10 (1982): 743–773. 6753756 10.1001/archderm.118.10.743

[2] R. M. Bhat , “Pyoderma Gangrenosum: An Update,” Indian Dermatology Online Journal 3, no. 1 (2012): 7–13. 23130252 10.4103/2229-5178.93482 PMC3481925

[3] A. Alavi , L. E. French , M. D. Davis , A. Brassard , and R. S. Kirsner , “Pyoderma Gangrenosum: An Update on Pathophysiology, Diagnosis and Treatment,” American Journal of Clinical Dermatology 18, no. 3 (2017): 355–372. 28224502 10.1007/s40257-017-0251-7

[4] E. Maverakis , C. Ma , K. Shinkai , et al., “Diagnostic Criteria of Ulcerative Pyoderma Gangrenosum: A Delphi Consensus of International Experts,” JAMA Dermatology 154, no. 4 (2018): 461–466. 29450466 10.1001/jamadermatol.2017.5980

[5] T. Brooklyn , G. Dunnill , and C. Probert , “Diagnosis and Treatment of Pyoderma Gangrenosum,” BMJ 333, no. 7560 (2006): 181–184. 16858047 10.1136/bmj.333.7560.181 PMC1513476

[6] U. Wollina , “Pyoderma Gangrenosum – A Review,” Orphanet Journal of Rare Diseases 2, no. 1 (2007): 19. 17433111 10.1186/1750-1172-2-19 PMC1857704

[7] D. S. Mowlds , J. J. Kim , P. Murphy , and G. A. Wirth , “Pyoderma Gangrenosum: A Case Report of Bilateral Dorsal Hand Lesions and Literature Review of Management,” Canadian Journal of Plastic Surgery = Journal Canadien de Chirurgie Plastique 21, no. 4 (2013): 239–242. 24497766 PMC3910530

[8] S. Reynolds and S. Smith , “Recurrent Bilateral Lower Leg Ulcers in an Adult Patient: A Case Report,” Journal of Case Reports 2 (2016): 1.

[9] A. Flora and J. W. Frew , “Pyoderma Gangrenosum: A Review of the Clinical, Mechanistic and Therapeutic Landscape,” Wound Practice and Research 30, no. 1 (2022): 16–23, https://journals.cambridgemedia.com.au/wpr/volume‐30‐number‐1/pyoderma‐gangrenosum‐review‐clinical‐mechanistic‐and‐therapeutic‐landscape.

[10] W. Xin , L. Gu , F. Du , T. Li , and S. Ye , “Case Report: Spesolimab for Pyoderma Gangrenosum in an Undifferentiated Oligoarthritis Patient Receiving Anti‐IL‐17 Therapy,” Frontiers in Immunology 16 (2025): 1581996, 10.3389/fimmu.2025.1581996/full. 40260260 PMC12009905

[11] H. Zhang , C. Wu , and H. Jin , “Successful Treatment of Etanercept‐ and Adalimumab‐Resistant Pyoderma Gangrenosum With Spesolimab, Moderate‐Dose Corticosteroids, and Minocycline,” Journal of Dermatological Treatment 36, no. 1 (2025): 2451811. 39828267 10.1080/09546634.2025.2451811

[12] K. Kohandel , S. Ala , B. Tamizifar , M. Karaminia , and M. Sahraian , “Pyoderma Gangrenosum in a Patient With Multiple Sclerosis Under Natalizumab Treatment: A Case Report,” BMC Neurology 25, no. 1 (2025): 137. 40175973 10.1186/s12883-025-04146-z PMC11963391

[13] R. A. Shamleh , A. Souriti , P. Keating , and N. Das , “Pyoderma Gangrenosum of the Abdominal Wall Skin in a Postoperative Gynaecologic Oncology Patient,” BMJ Case Reports 18, no. 8 (2025): e266931. 10.1136/bcr-2025-266931 40759505

[14] F. Alassani , P. Kassang , E. G. Amouzou , et al., “Paraneoplastic Pyoderma Gangrenosum Associated With Adenocarcinoma of the Rectosigmoid Junction: A Case Report,” Journal of Medical Case Reports 7, no. 13 (2019): 372. 10.1186/s13256-019-2290-6 PMC6898921 31810486

[15] M. Shah , M. Sachdeva , A. Gefri , and A. Jfri , “Paraneoplastic Pyoderma Gangrenosum in Solid Organ Malignancy: A Literature Review,” International Journal of Dermatology 59, no. 2 (2020): 154–158. 31512760 10.1111/ijd.14637

[16] X. Q. Zhang , Z. W. Tang , and J. Jing , “Progressive Facial Ulcer: A Case Report of Pyoderma Gangrenosum,” Journal of Inflammation Research 17 (2024): 687–691. 38332897 10.2147/JIR.S441751 PMC10849904

[17] F. Wang , L. Li , W. Li , et al., “Clinical Characteristics, Treatment, and Wound Management of Pyoderma Gangrenosum: A Case Series,” PLoS One 20, no. 6 (2025): e0326203. 40549753 10.1371/journal.pone.0326203 PMC12185014

